# Mechanisms Underlying Aggressive Behavior Induced by Antiepileptic Drugs: Focus on Topiramate, Levetiracetam, and Perampanel

**DOI:** 10.1155/2018/2064027

**Published:** 2018-11-15

**Authors:** Cerine C. Hansen, Hanna Ljung, Eylert Brodtkorb, Arne Reimers

**Affiliations:** ^1^Faculty of Medicine and Health Sciences, Norwegian University of Science and Technology, Trondheim, Norway; ^2^Department of Neurology and Rehabilitation Medicine, Skåne University Hospital, Lund, Sweden; ^3^Department of Clinical Neurosciences Lund, Faculty of Medicine, Lund University, Lund, Sweden; ^4^Department of Neurology and Clinical Neurophysiology, St. Olavs University Hospital, Trondheim, Norway; ^5^Department of Neuromedicine and Movement Science, Norwegian University of Science and Technology, Trondheim, Norway; ^6^Department of Clinical Chemistry and Pharmacology, Skåne University Hospital, Lund, Sweden; ^7^Division of Clinical Chemistry and Pharmacology, Lund University, Lund, Sweden

## Abstract

Antiepileptic drugs (AEDs) are effective against seizures, but their use is often limited by adverse effects, among them psychiatric and behavioral ones including aggressive behavior (AB). Knowledge of the incidence, risk factors, and the underlying mechanisms of AB induced by AEDs may help to facilitate management and reduce the risk of such side effects. The exact incidence of AB as an adverse effect of AEDs is difficult to estimate, but frequencies up to 16% have been reported. Primarily, levetiracetam (LEV), perampanel (PER), and topiramate (TPM), which have diverse mechanisms of action, have been associated with AB. Currently, there is no evidence for a specific pharmacological mechanism solely explaining the increased incidence of AB with LEV, PER, and TPM. Serotonin (5-HT) and GABA, and particularly glutamate (via the AMPA receptor), seem to play key roles. Other mechanisms involve hormones, epigenetics, and “alternative psychosis” and related phenomena. Increased individual susceptibility due to an underlying neurological and/or a mental health disorder may further explain why people with epilepsy are at an increased risk of AB when using AEDs. Remarkably, AB may occur with a delay of weeks or months after start of treatment. Information to patients, relatives, and caregivers, as well as sufficient clinical follow-up, is crucial, and there is a need for further research to understand the complex relationship between AED mechanisms of action and the induction/worsening of AB.

## 1. Introduction

With a prevalence of about 0.6–0.7% in developed countries, epilepsy is the fourth most common neurologic disease after migraine, Alzheimer's disease, and stroke [1, 2]. Most patients receive treatment with antiepileptic drugs (AEDs), and up to 70% of them become seizure-free [3]. However, AEDs are potent agents that can induce numerous adverse reactions and drug-drug interactions. Psychiatric and behavioral adverse reactions (PBAR) are common. They include depression, anxiety, psychosis, and aggressive behavior (AB) [4]. In everyday practice, the numerous clinical expressions of AED-induced PBAR may be difficult to distinguish from endogenous clinical manifestations in the individual patient.

Levetiracetam (LEV), perampanel (PER), and topiramate (TPM) are currently identified as AEDs with the strongest evidence for AB. However, benzodiazepines, brivaracetam (BRV), phenobarbital, tiagabine, vigabatrin, and zonisamide are also associated with a higher occurrence of AB compared to other AEDs [4]. The risk is increased in patients with a previous history of psychiatric disorders [4–6]. This kind of adverse effect can become a significant clinical problem since these AEDs often are used in difficult-to treat epilepsy. When improved seizure control is achieved with these drugs, the occurrence of intolerable PBAR necessitating discontinuation of the effective drug is highly unfortunate.

It is unclear which pharmacological mechanisms evoke AB. Eventually, multiple mechanisms of action (MOAs) have been identified for most AEDs. Despite this, AEDs are usually classified according to their proposed “main” or “principal” MOA, although such categorization is of limited clinical value. This is illustrated by the observation that AEDs with different principal MOAs can have identical therapeutic effects, while AEDs with a similar principal MOA can have divergent therapeutic effects. Likewise, AEDs with different principal MOAs can induce identical adverse effects, while AEDs with an identical principal MOA may have different safety profiles.

LEV, PER, and TPM have divergent pharmacological profiles with several different MOAs. Yet, they can all induce AB. While LEV and PER have been assigned a principal MOA, TPM has been actively marketed as a “multiple-MOA” AED.

These three main culprit drugs will be used as models to discuss established knowledge as well as various hypotheses about AB as an adverse effect of AEDs. Three main questions will be addressed:

1. Which MOAs can induce AB?
2. Do these AEDs (LEV, PER, and TPM) have a common MOA that is responsible for this particular adverse effect?
3. Could AB be an indirect effect, i.e., the consequence of the clinical efficacy of these AEDs?

This review is based on searches in various online repositories (PubMed, ResearchGate, Google Scholar, and EMBASE) using «antiepileptic drugs», «levetiracetam», «perampanel» and «topiramate», combined with terms such as «behavior», «psychiatric side effects», «aggression», «agitation», «irritability», and «adverse effect». The searches included publications until February 2018.

## 2. Aggressive Behavior: Epidemiology, Etiology, and Treatment

It is well-documented that the prevalence of psychiatric conditions is higher in people with epilepsy than in the general population. It is estimated that as much as 30% of newly diagnosed and 50% of treatment-resistant patients have a psychiatric disorder, mainly depression, anxiety, and psychosis [7]. It may therefore be assumed that AB is common in people with epilepsy. However, the actual prevalence is not known [8].

Aggression is a social behavior that is aimed at eliciting discomfort, pain, or physical damage, to oneself, to another person, or to things or at defending oneself against a threat. AB can be defensive, instrumental (planned with the intention of achieving a goal), or impulsive (in anger and after provocation) [4].

AB can occur as a symptom of various medical conditions such as brain damage, encephalitis, drug use, dementia, intoxication, psychosis, affective disorders, and personality disorders as well as in relational, behavioral, developmental, and adaptational disorders [9]. This implies that AB occurs not only as a permanent personality trait but also as a temporary behavior change. It is estimated that up to 60% of people with intellectual disability exhibit signs of AB [10].

The heterogeneity of AB suggests a complex etiology [11]. Indeed, AB has been associated with genetic, epigenetic, neurobiological, and psychosocial factors [12]. Several cortical and subcortical brain networks are involved, predominantly those mainly modulated by the monoamines serotonin (5-HT), dopamine (DA), and norepinephrine (NE), but also glutamate and gamma-amino-butyric acid (GABA) play an important role. Dysregulation of several proteins in these networks contribute to AB. These include 5-HT_1A_ and 5-HT_2A_ receptors, 5-HT transporters, DA D_1_ and D_2_ receptors, DA transporters, *α*1 and *α*2 adrenoceptors, monoaminoxidase (MAO) A, GABA_A_ and GABA_B_ receptors, GABA transaminase, glutamatergic N-methyl-D-aspartate (NMDA), and *α*-amino-3-hydroxy-5-methyl-4-isoxazolepropionic acid (AMPA) receptors, as well as voltage-regulated sodium and calcium channels [13, 14].

Other neuroactive substances may also interact with these networks, e.g., steroid hormones, vasopressin, histamine, substance P, nitrogen monoxide (NO), neural cell adhesion molecule (NCAM), and interleukins [14]. Imaging studies have identified brain structures that are associated with AB, such as the prefrontal cortex, amygdala, hypothalamus, hippocampus, septal nuclei, and periaqueductal gray matter (PAG) [12].

Treatment of AB is versatile, including drugs and nonpharmacological interventions. Because of the diverse and complex etiology, as well as different comorbidities, the choice of intervention and type of drug treatment may vary considerably between individual patients. AB in conjunction with acute psychosis or mild depression, for instance, needs different treatment approaches [11]. A plethora of drugs may be used to treat AB. Second-generation antipsychotic drugs have been used, based on their ability to modulate several receptors involved in AB, such as 5-HT, DA, NMDA, NE, and GABA receptors [13]. Benzodiazepines, being allosteric agonists at GABA_A_ receptors, have also been used. However, they may elicit paradoxical reactions, i.e., reinforced AB [12]. Selective serotonin reuptake inhibitors (SSRI), *β*-adrenergic blockers, psychostimulants (e.g., amphetamine), lithium, and AEDs like valproate, lamotrigine, gabapentin, and TPM have all been shown to be effective [8, 13]. Nevertheless, the most promising treatments will be those that take underlying, specific processes into consideration [11].

## 3. Aggressive Behavior as an Adverse Effect of AEDs

It has been estimated that up to 50% of AED users experience adverse reactions, leading to discontinuation of the culprit drug in up to 20% of all cases [15–17]. Generally, most newer AEDs have better tolerability profiles than the older ones [17]. Many adverse effects are dose-dependent and often involve the central nervous system, such as dizziness, sedation, ataxia, nystagmus, and impaired cognitive functions.

AEDs may frequently induce PBAR, including depression, anxiety, psychosis, and AB. The prevalence of such adverse effects in adults with epilepsy has been estimated to be 8–20% [4, 18] and 11–14% in patients ≤ 18 years [19]. It can be difficult to distinguish between psychiatric adverse effects that are induced by AEDs and preexisting traits that are worsened by AEDs, since such conditions are common in people with epilepsy [20]. LEV, PER, and TPM are associated with the highest reported frequency of AB among AEDs, particularly in patients with a previous history of psychiatric symptoms [4, 20, 21]. The recently introduced BRV, which is chemically closely related to LEV, is said to have less potential to induce behavioral side effects than LEV [6, 22, 23]. However, no studies that directly compare LEV and BRV have been published. In children and adolescents, there is also an increased risk of AB associated with gabapentin, phenobarbital, valproate, and zonisamide [4]. Predisposing endogenous factors are previous psychiatric condition, frontal lobe epilepsy, absence epilepsy, and difficult-to-treat (“treatment-resistant”) epilepsy [19].

Table 1 provides an overview of various PBAR of LEV, PER, and TPM and their frequencies. Aggression and irritability are categorized as “common” adverse effects in their respective summary of product characteristics (SPC), meaning that they occur with a frequency of 1–10% [24–26]. Some studies report even higher frequencies, e.g., up to 16% for LEV [27]. TPM on the other hand shows the broadest spectrum of PBAR, including anxiety, agitation, aggression, depression, and psychosis [28]. The SPC for BRV states irritability as common and aggression as uncommon [29]. However, newer studies report higher frequencies, although still lower than for LEV [5, 6].

It is difficult to predict at which point in time PBAR will become manifest, since data from clinical studies are scarce and not uniform (Tables 2–4). Most studies merely report that PBAR occurred during the study period, and only a few studies state a time interval from start of treatment until the adverse effect emerged. Dinkelacker et al. [30] report an interval of 3.6 months from start with LEV to the recognition of PBAR. Similarly, Mula et al. [31] report an average delay of 88 days for mainly aggression, agitation, anger, and hostile behavior. Other studies state a much shorter interval of less than one month [32, 33]. For PER, various time intervals have been reported: within six weeks [34], three months [35, 36], or even six months [36, 37]. For TPM, Mula et al. [38] state an interval of 60 days for the emergence of affective disorders and aggression, even later for psychosis. However, it is difficult to sort out to what extent the delayed reactions might be associated with a gradual dose increase.

People with epilepsy seem to be more susceptible to PBARs from AEDs, particularly LEV and PER, since the prevalence of such reactions is lower when these drugs are used for non-epilepsy conditions (Tables 2 and 3) [4, 21]. Moreover, some data suggest that the incidence and clinical characteristics of AB depend not only on previous psychiatric history but also on age, sex, type of epilepsy, and AED dose [28]. This is discussed in Section 5.

Adverse reactions involving the CNS are often, but not always, dose-dependent, and it seems that the risk for PBAR can be reduced by low initial doses and slow titration [39–42]. This applies particularly to PER, since many studies found that adverse effects primarily occur with doses of 8 or 12 mg/day. In phase III clinical studies, the overall rate of psychiatric TEAEs was 17.2% (8 mg) and 22.4% (12 mg) (placebo: 12.4%) [34, 43–48]. Regarding LEV, the literature is more diverse. Some studies suggest that adverse reactions to LEV are mostly dose-independent, as they may occur at any dose and despite slow titration, while others found that the likelihood of LEV being discontinued or lowered was higher when it was initiated at a high dose [49–53]. With TPM, slow titration may reduce the risk, although adverse reactions may occur at any dose. PBAR induced by TPM usually resolve upon dose reduction [38, 50, 54–56].

## 4. Possible Neuropharmacological Mechanisms of AED-Induced Aggressive Behavior

### 4.1. Levetiracetam

Levetiracetam (LEV) is effective in focal onset seizures as well as in generalized onset tonic-clonic and myoclonic seizures [24]. LEV is a pyrrolidone derivative that has been developed from piracetam. It is presumed to act on presynaptic neurotransmitter release by binding to synaptic vesicle protein 2A (SV2A), a glycoprotein that is part of the membrane of presynaptic neurotransmitter-containing vesicles in neurons and neuroendocrine cells. SV2A and related isoforms (SV2B, SV2C) are expressed in several locations in the brain, especially in the cortex but also in subcortical regions such as thalamus, basal ganglia, and hippocampus. Reduced expression of SV2A may lead to a lower seizure threshold and epileptogenesis [84].

It is not clear exactly how LEV's binding to SV2A results in antiepileptic efficacy, but it is assumed that this protein is involved in exocytosis of neurotransmitters and that this exocytosis is downregulated either via reduced calcium inward currents or other modulating mechanisms [85]. The recently introduced AED, BRV, is a derivative of LEV/piracetam and has a higher affinity to SV2A, although it has already been shown that BRV also acts as a sodium channel blocker [86].

LEV also increases tissue concentrations of GABA, neutralizes the action of negative modulators of the GABA_A_ receptor, and reduces the excitatory action of glutamate by modulation of AMPA receptors [84, 87–92]. Several studies suggest that LEV modulates neuronal cell function via additional pharmacological mechanisms including modulation of serotonergic and *α*2-adrenergic signaling paths as well as *μ*-opioid receptors [93]. LEV also modulates intraneuronal calcium levels via inhibition of N-type calcium channels. Other MOAs associated with LEV are modulation of presynaptic P/Q-type calcium channels and potassium channels, as well as upregulation of glutamate transporters in glial cells [84, 91, 94]. It is not clear whether these MOAs occur on their own or as a consequence of the interaction with SV2A [84, 93].

The broad pharmacological effect of LEV makes it difficult to determine the exact cause of AB. The high rate of AB with LEV may not necessarily be related to SV2A, since it has been suggested that BRV, which has a 15–30 times higher affinity to SV2A than LEV, is associated with a lower incidence of AB than LEV [6, 22, 23, 95]. Interestingly, it seems that BRV does not modulate NMDA, AMPA, or kainate receptors [96, 97]. These findings suggest that LEV's negative modulating effect on AMPA receptors contributes to increased AB. This idea is supported by the observation that piracetam (the predecessor of LEV) is not associated with increased AB. Piracetam improves neural and cognitive functions, presumably via positive allosteric modulation of the AMPA receptor [98, 99]. The interaction between NMDA and AMPA receptors and AB is discussed in more detail under Section 4.2.

5-HT (serotonin) and GABA have also been associated with AB [4, 32, 42, 100]. 5-HT is possibly the best-studied neurotransmitter in relation to AB, especially impulsive aggression [4, 12, 100, 101]. Several studies suggest that 5-HT modulates brain activity in the prefrontal cortex, which controls limbic system responses to stimuli, i.e., regulation of emotions. It has been speculated that reduced levels of 5-HT and its metabolite 5-hydroxyindoleacetate (5-HIAA) are associated with impulsive aggression [101, 102]. However, the relationship between 5-HT and behavior is complex [4, 101]. The 5-HT-system consists of at least 14 different receptors with subtypes, both pre- and postsynaptic, with unique and partly antagonistic effects on aggression [4, 101]. Undoubtedly, 5-HT is involved in AB, but whether LEV might interfere with this mechanism is unclear. The relationship between GABA and AB is discussed under Section 4.3.

### 4.2. Perampanel

Perampanel (PER) is licensed as add-on treatment for focal onset seizures and generalized onset tonic-clonic seizures in patients > 12 years [25]. It acts as a highly selective, noncompetitive antagonist on AMPA receptors, thereby reducing glutamatergic transmission. In contrast to competitive antagonists, noncompetitive antagonists will not be overcome by high synaptic glutamate concentrations. PER reduces calcium inward currents through AMPA receptors in cortical and subcortical brain regions. Some data suggest that it also acts on NMDA and kainate receptors [103]. PER is one of the newest AEDs, and presently, there is no evidence that it acts on other pharmacological targets.

Increased levels of glutamate are associated with increased AB, particularly impulsive aggression [4, 12, 104]. This is believed to be mediated by stimulation of glutamatergic receptors in the amygdala, hypothalamus, and periaqueductal gray matter [104]. Genetic modification of AMPA and NMDA receptors in mice leads to changes in AB [4, 104–106]. However, glutamate's effect on behavior is complex and studies demonstrated that blocking of AMPA receptors can both decrease and increase AB [106, 107]. It has been demonstrated that phencyclidine, a NMDA antagonist, increases aggression at low doses, but decreases it at higher doses [108].

### 4.3. Topiramate

Topiramate (TPM) is effective against focal onset seizures and generalized onset tonic-clonic seizures [26, 109]. Additionally, it is effective as a prophylactic treatment of migraine [26, 109]. Topiramate has several MOAs. While none of them has been pointed out as the principal MOA, three of them have received most attention: blockade of voltage-dependent sodium and calcium channels, enhancement of GABA-dependent chloride inward currents, and antagonism at glutamatergic AMPA and kainate receptors [26, 109, 110]. These channels and receptors are all involved in aggressive behavior [4]. TPM also inhibits carbonic anhydrase types II and IV, although this MOA is not believed to contribute noteworthy to TPM's antiepileptic effect [26, 110]. Some studies have shown that TPM has neuroprotective properties [111]. Being a fructose derivative, TPM is structurally unrelated to other AEDs (although it shares with zonisamide a sulfamate group) [26, 109, 110].

### 4.4. One Common Mechanism?

Having reviewed the different pharmacological profiles of LEV, TPM, and PER, it is still not possible to conclude with certainty which MOA is responsible for the increased rate of AB in people treated with these drugs. Available data suggest that 5-HT, glutamate, and GABA play a major role in AB. Since all three AEDs have an inhibiting effect on glutamatergic transmission via the AMPA receptor, it appears most promising for future research to focus on this mechanism [18]. One caveat is that these MOAs are only the ones that we are currently aware of, but this may change. It cannot be ruled out that LEV, PER, and TPM exert part or most of their therapeutic and undesired effects via other MOAs that have not been discovered yet.

## 5. Biological Vulnerability

A wide range of clinical factors may interact to lay the ground for the development of AB induced by AEDs.

### 5.1. The Epileptic Disorder Itself

Neurological and psychiatric conditions may generally increase the vulnerability for PBAR [67]. This is in line with the observation that the rate of PBAR is lower in patients using AEDs for non-epilepsy conditions [4, 21]. It has been speculated that the increased vulnerability is due to structural and functional cerebral alterations.

Generalized onset seizures, particularly absence seizures, are associated with an increased risk of psychiatric and behavior-related symptoms, including anger, irritability, and aggression [18, 19, 24, 53]. It has been suggested that absence seizures have a cortical origin in the frontal lobe and involve the thalamus which may cause general functional impairment. These brain regions are associated with regulation of aggressive behavior [4, 18, 19, 112].

Juvenile myoclonic epilepsy (JME) is the most common form of idiopathic generalized epilepsy. It is associated with personality disorders, psychosocial maladjustment, and psychiatric comorbidity including substance and alcohol abuse [113, 114]. Impulsiveness, quick and frequent mood changes, and risk-seeking behavior are reported in a subset of these patients [114]. Executive functions, e.g., problem-solving, planning, execution of tasks, and behavioral control, are often impaired. This has been associated with frontal lobe dysfunction, as suggested by neuropsychological testing and advanced imaging [113, 114]. It seems that patients with JME are more vulnerable for PBAR induced by AEDs [113]. However, the clinical heterogeneity is pronounced, and psychosocial outcome and treatment responses vary widely in JME [114].

Besides generalized epilepsy, temporal lobe epilepsy (TLE) as well is associated with psychiatric symptoms, including aggression [4]. The medial part of the temporal lobe contributes to the regulation of emotions by its connection to the limbic system. Structural or functional abnormalities in the medial temporal lobe, like neuronal loss, synaptic reorganization, or changes in the hippocampus or the amygdala, are associated with a disposition for the development of AB [4, 34, 115]. A previous history of febrile seizures or status epilepticus is often involved [4, 67, 115]. Brodie et al. [4] suggest that the structural changes seen with TLE may lead to growth of immature GABAergic neurons that convey excitation instead of inhibition, as seen in the brain of newborns. Hence, AEDs that reinforce GABA, i.e., LEV or TPM, would increase neuronal excitement instead of decreasing it [4]. Similar paradoxical effects may take place in the glutamatergic system, which implies that AEDs that normally inhibit glutamatergic signal transmission (LEV, PER, and TPM) might instead have a facilitating effect [4]. How these changes might affect the propensity to PBAR is not clear.

### 5.2. Psychiatric Comorbidity

The relationship between structural anomalies in the brain and PBAR is further illustrated by the fact that AB is frequently seen in patients with central nervous pathology, e.g., due to trauma or infection [116]. The concept of the interictal dysphoric disorder means that patients with epilepsy may exhibit the following psychiatric symptoms between seizures: depressed mood, reduced energy, pain, insomnia, anxiety, mood swings, and outbursts of irritability and AB irritability [117]. Patients with epilepsy may also present atypical behavioral symptoms that occur peri-ictally, i.e., before, during, or after an epileptic seizure [32, 117]. Prodromal and immediate postictal symptoms often manifest with dysphoric, emotional, and behavioral symptoms [118]. Postictal psychosis is a potentially dangerous complication of chronic epilepsy usually occurring with a lucid interval within one week after a cluster of (usually tonic-clonic) seizures. It may be associated with religious, paranoid, and persecutory ideas causing pronounced aggressive behavior [119]. A case of homicide was recently reported during postictal psychosis and was thought to be promoted by a preceding treatment switch from carbamazepine to LEV [120]. Furthermore, psychiatric symptoms that emerge after seizure control may represent an entity on its own, called “alternative psychosis” (see chapter 6.3). The above-mentioned phenomena illustrate how difficult it can be to distinguish between AED-induced PBAR and endogenous as well as seizure-related psychiatric and behavioral symptoms.

### 5.3. Genetic Influence

Since patients with difficult-to-treat epilepsy and a personal or family history of psychiatric disorders have a higher risk of PBAR, the question of a genetic predisposition has been discussed [4, 18, 67, 68]. Recently, numerous copy number variations have been uncovered as important risk factors for the development of multiple neuropsychiatric disorders [121]. Such chromosomal rearrangements may underlie a broad phenotype spectrum, ranging from normal development to mild learning- or intellectual disabilities, epilepsy, and psychiatric diseases, such as autism spectrum disorders and schizophrenia, often in combination [122–124]. The epilepsy is frequently of generalized type [121]. Conceivably, this vulnerable group of patients may harbor a particular susceptibility to develop complex PBAR from AEDs. Moreover, an association study by Helmstaedter et al. investigated LEV as a model AED for PBAR and found several genetic polymorphisms that are associated with reduced dopaminergic activity in patients having the most pronounced reactions [125]. However, as there are no further such studies, it is not clear whether these findings apply to other AEDs besides LEV [4, 125].

### 5.4. Intellectual Disability

From a lifetime perspective, people with intellectual disability are among the most drug-exposed groups in society. Epilepsy is the most common comorbidity in these individuals. They may not be able to report and describe adverse reactions from AEDs in the form of slowing of central information processing (114). Symptoms of overdosing, such as sedation, ataxia, or blurred vision, may even occur unnoticed by the caregivers [68, 84, 126]. Such unspecific adverse reactions are not uncommon with LEV, PER, and TPM (Table 1) and may be indirectly expressed as disturbed behavior and interpreted as specific pharmacodynamic effects [57, 127, 128]. It is also well-known that sedating drugs can paradoxically induce hyperactivity, especially in children [57]. TPM, in addition, can impair language function and reduce verbal fluency [128, 129]. This may be more pronounced in patients with lower educational levels, suggesting an impact of baseline cerebral performance [129]. Impaired ability to express oneself may trigger AB. Moreover, these patients often use AED polytherapy and other drugs targeting the brain, which may cause pharmacodynamic interactions and further increase the risk of disturbed behavior [28, 115].

In contrast, the “release phenomenon” denotes challenging conduct in patients disabled by a previously severe drug-resistant seizure disorder who obtain seizure control with newer drugs with less impact on alertness and cognition. This occurs usually in patients with intellectual disability, who may express increased vigilance and self-assertion as AB. A more demanding behavior should not invariably be interpreted as a sign of drug toxicity [114].

## 6. Other Potential Mechanisms

### 6.1. Hormonal and Biochemical Aspects

Various steroid hormones modulate AB, and studies have shown an association between high CNS levels of testosterone and impulsive-aggressive behavior [14, 130–132]. Testosterone may interact with the serotonin system and increase neuronal activity in brain regions involved in AB, such as the amygdala, hypothalamus, and periaqueductal gray matter (PAG) [130, 131]. Low levels of serotonin together with high levels of testosterone seem to play an important role in aggression [130]. Synthetic testosterone analogues have been shown to alter the expression of GABA_A_ and DA receptors and increase levels of vasopressin, substance P, and stress hormones [133]. Not surprisingly, aggressive behavior is much more frequently seen in male than in female patients with epilepsy [134, 135]. However, while women show less aggression, they tend to be more irritable than men [136].

It has been suggested that LEV inhibits aromatase, an enzyme that converts testosterone to estradiol [137, 138]. This would imply that patients using LEV may have higher levels of testosterone (and, possibly, reduced levels of estradiol). This could, at least partially, explain the increased prevalence of AB in patients using LEV. Birger et al. (2003) demonstrated that administration of testosterone in rats increased the expression of 5-HT_2A_ receptors and other 5-HT binding sites and that this most probably was an effect mediated by estradiol [130]. Inhibition of aromatase by LEV could therefore produce a dual negative effect on the serotonin system: increased testosterone levels may downregulate 5-HT, and decreased estradiol produces fewer 5-HT receptors and binding sites.

Stress is a trigger for both epilepsy and psychiatric disorders, and there is a significant overlap of the neural networks involved in stress and aggression [139, 140]. It is possible that AEDs directly or indirectly affect those hormones of the hypothalamus-pituitary-adrenal gland axis that are involved in regulation of stress responses [139].

Brodie et al. [4] point out that TPM, a carbonic anhydrase inhibitor, can induce metabolic acidosis, which is associated with aggression and irritability [4]. Interestingly, this pharmacologic characteristic is shared by zonisamide, an AED that is also associated with an elevated risk of PBAR [18].

### 6.2. Epigenetics

Epigenetics explains how dynamic environmental factors can affect the expression of genes and the pathophysiology of disease states without changing the genetic code [141]. In recent years, much attention has been directed toward AEDs and their impact on crucial epigenetic processes such as histone acetylation and DNA methylation [4, 12, 142]. Histones are proteins that are bound to the DNA. Their acetylation state affects the accessibility of the DNA and, thus, gene transcription and expression [142]. Acetylation is controlled by two enzymes called histone acetyltransferase (HAT) and histone deacetylase (HDAC). While little is known about the exact mechanisms, an association between HDAC and behavior has been found, including AB [142].

Valproate, a broad-spectrum AED and a mood stabilizer, possesses several MOAs, including inhibition of HDAC [4, 12, 13, 142, 143]. This contributes to increased expression of reelin and GAD67 in cortical GABAergic interneurons which may reduce aggression, as downregulation of reelin and GAD67 has been observed in patients with schizophrenia and bipolar disorder. These patients often show more anger and aggression than the general population [12, 142]. It has also been found that TPM and the main metabolite of LEV inhibit HDAC, but for now little is known how that may affect AB [143].

Further epigenetic mechanisms associated with AEDs and aggression are modulation of the serotonin system in the amygdala and the prefrontal cortex, as well as monoaminoxidase A activity [4, 142]. By now, it is not known whether PER exerts epigenetic effects.

### 6.3. Forced Normalization and Alternative Psychosis

“Forced normalization” (FN) is an EEG phenomenon [32, 115] that was first described by Landolt in 1953. He observed that patients with epilepsy developed psychiatric symptoms, mainly psychosis, when their EEG became normal and seizure control was achieved [144]. In 1965, Tellenbach introduced the term “alternative psychosis” which is the clinical counterpart of FN [115]. Later, “alternative” phenomena have been expanded to include other psychiatric symptoms as well, e.g., depression, anxiety, hypomania/mania, and aggression [4, 115, 145]. Hence, it is possible that the psychiatric adverse reactions seen with AEDs not necessarily are direct pharmacological effects, but sometimes a neurophysiological consequence of improved seizure control.

Although the concept of FN/alternative psychosis was long ago acknowledged, its underlying mechanisms are essentially unknown [56, 146, 147]. It is thought to be related to the antagonism between epilepsy and psychosis, as epileptic seizures occasionally abort psychiatric symptoms (which also is the rationale for treating psychiatric conditions with electroconvulsive therapy) [148]. It has been speculated that some patients with epilepsy have a preexisting imbalance of neurotransmitters that would cause psychiatric symptoms would they not be prevented by recurrent epileptic seizures that lead to stabilization. A related possible explanation is the kindling phenomenon, where repeated stimulation of the limbic system, mainly the amygdala, is supposed to induce behavioral changes [146, 147, 149].

It has been reported that alternative psychosis occurs in relation to the introduction of new AEDs, and both LEV and TPM are examples [41, 67, 146, 149]. It is, however, important to understand that alternative psychiatric symptoms are not limited exclusively to drug treatment as it also may occur when seizure control is achieved by other methods, e.g., surgery [42, 115, 147]. From this, it follows that this clinical phenomenon does not depend on one distinct pharmacologic mechanism [32, 67]. Moreover, the concept of FN/alternative psychosis alone does not fully explain AB with AED use, since several studies have shown that PBAR also occurs in patients who do not become seizure-free [28, 32, 67]. Some studies also report that AB may be associated with deteriorated seizure control, which again illustrates the complex relationship between epileptic activity and behavior [56]. In clinical practice, it is important to clarify if psychiatric symptoms in patients using AEDs are adverse drug reactions, a consequence of seizure control, seizure breakthrough or an expression of a more complex, endogenous aptness for psychiatric disorders [4, 67].

### 6.4. Aggression Induced by Other Drugs

To identify possible mechanisms by which AEDs may induce AB, it could be useful to look at other drugs that also have the potential to induce this adverse reaction. Interestingly, several drugs used to treat aggression have been reported to induce AB. Among those are benzodiazepines, antidepressants, central stimulants [150–152], and AEDs, among them TPM [153].

Benzodiazepines increase the inhibitory actions of GABA via allosteric modulation of the GABA_A_ receptor, thereby increasing its affinity for GABA [12, 150]. While most adverse reactions to sedative drugs are predictable, some patients may develop paradoxical reactions such as increased irritability, aggression, hostility, and impulsivity. Usually, this occurs in children, in elderly patients, and in patients with intellectual disability [150]. The paradoxical reactions are presumably due to disinhibition of behavioral networks that normally are balanced. This is based on the theory that GABA plays a role in AB, yet it is speculative [4, 150]. It has been found that the risk of AB is doubled in children and adolescents using antidepressants (SSRI, SNRI) that increase the amount of 5-HT and NA in synaptic clefts [151]. These monoamines are involved in AB [4]. Among central stimulants, particularly amphetamine and its derivatives are associated with irritability [152]. Amphetamines both increase the release and inhibit the reuptake of NE and DA in the synapse. In higher doses, they also inhibit 5-HT. High levels of NA and DA and low levels of 5-HT have been suggested to promote aggression and irritability [4, 152].

Other drugs that can induce AB are antihistamines, statins, and anabolic steroids [154–156]. In children, second-generation antihistamines can produce aggression, agitation, and hyperactivity [154]. Antihistamines act primarily as antagonists at the histamine H1 receptor. As mentioned above, low levels of 5-HT may promote AB, and it has been shown that histamine and H1 receptors in the brain can modulate AB via the 5-HT system [14]. Statins are another class of drugs that may induce increased irritability, which suggests a relationship between lowered cholesterol and AB [155]. These drugs are commonly used in combination with AEDs in elderly patients with vascular epilepsy.

It is not surprising that AB is a common adverse reaction to anabolic-androgenic steroids (AAS) [133, 156, 157]. Studies have shown that AAS not only increase AB temporarily, but also may lead to psychiatric long-term consequences as their use in or close to puberty may induce permanent changes in the developing brain [133, 156, 157]. AAS has been shown to modify the expression of cerebral androgen, GABA_A_, and DA receptors, as well as affect the 5-HT system and the levels of neuroactive substances, e.g., vasopressin, substance P, and stress hormones [133]. Carrillo et al. found that AAS reinforce glutamatergic connections between the hypothalamus and the stria terminalis. Their study supports that glutamate and vasopressin are involved in AB [158].

This review of AB induced by drugs that are not AEDs reveals some pharmacological similarities: (1) the modulation of GABAergic neurotransmission, demonstrated for both LEV and TPM and (2) inhibition of glutamatergic neurotransmission, particularly via the AMPA receptor—this has been demonstrated for LEV, PER, and TPM—and (3) modulation of the 5-HT system, which has been shown for LEV. Possible effects of AEDs on androgen and DA receptors as well as on neuroactive substances are poorly studied, but this does not mean that they do not exist. It must also be kept in mind that PER is one of the newest AEDs on the market. Chances are good that it may have pharmacological properties that have not yet been discovered. Likewise, all other drugs discussed here including LEV and TPM may possess unknown MOAs that contribute to their clinical effects.

## 7. Future Perspectives

Since little is certain and much is speculative regarding AB associated with AED treatment of epilepsy, and since it represents a significant clinical problem, further study on this topic is desirable. Studies on the pharmacological MOAs of AEDs and how they are related to AB would be particularly useful. This includes the search for yet unknown MOAs. New technologies like pharmacological magnetic resonance imaging (phMRI) may help to identify the sites of AED action in the brain [159]. This could be related to what is known about the etiology and the pathophysiology of AB. As LEV, PER, and TPM share an inhibiting effect on glutamatergic transmission via the AMPA receptor, the latter may represent a promising starting point [18]. Possible AED effects on hormones like testosterone, oxytocin, and stress hormones as well as on neuroactive substances like vasopressin or substance P deserve further research, e.g., by concentration measurement in CSF or brain tissue. The relation between epigenetic factors and AB is another promising area of future research [4, 142]. It is also desirable to develop instruments and clinical routines that help clinicians to define whether psychiatric symptoms in the individual patient are an adverse reaction to AEDs, a consequence of achieved seizure control, the seizure disorder itself and its underlying cause, or the manifestation of endogenous psychiatric conditions [4, 67]. Moreover, further clinical research attempting to identify vulnerability factors may be helpful in order to minimize the incidence of these drug effects.

## 8. Summary and Conclusion

LEV, PER, and TPM are associated with a higher risk of AB than other AEDs. They have various pharmacological MOAs, some of which interfere with neurotransmitters involved in AB. However, it is not clear which of them is the main one responsible for the increased prevalence of AB. In this context, it is important to note that the MOAs we know of today do not necessarily represent the complete and final spectrum of pharmacological effects of these drugs. Future research might unveil additional MOAs. There are indications that particularly 5-HT, glutamate, and GABA are involved in aggression, and the AMPA receptor looks like the most promising target. Other mechanisms by which drugs may induce AB include modulation of testosterone levels and of various neuroactive substances. Little is known about the role of epigenetics in aggression, but it has already been shown for some AEDs that they do interact with epigenetic mechanisms such as histone acetylation and DNA methylation.

**Figure 1:**
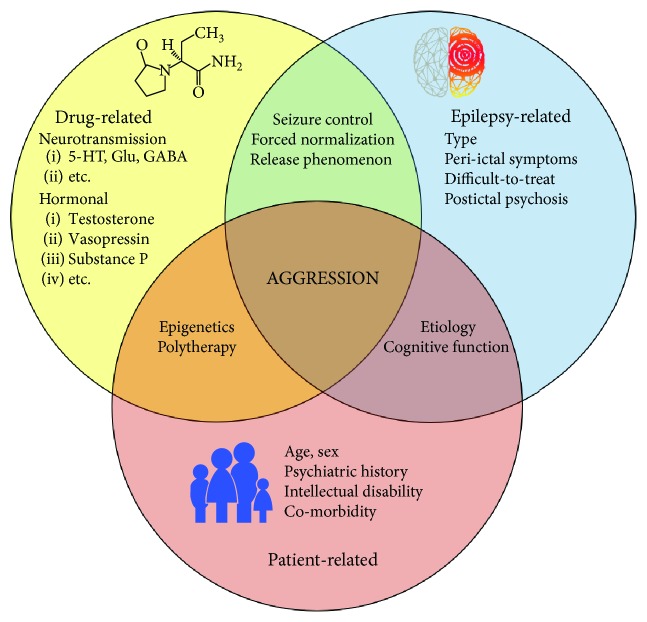
Summary of factors involved in aggressive behavior associated with antiepileptic drug treatment of epilepsy.

The biological vulnerability to PBAR from AEDs is multifaceted. A range of mechanisms and clinical predisposing factors may interact, including the phenomenon of alternative psychosis.  Figure 1 illustrates the complex and multifactorial background of AB in people with epilepsy. Drug related, epilepsy-related, and patient-related elements must be carefully evaluated in each case. Challenging behaviors from non-AED-related causes should be excluded. Consideration of the epilepsy type and etiology and the previous personal or familial psychiatric history should receive particular attention. A low total drug burden and a slow dose titration are prerequisites for best possible risk reduction. Remarkably, PBAR may first be recognized clinically several weeks or months after starting the culprit drug. Of utmost importance is information to the patients, relatives, or caregivers about potential PBAR, and the possibility of their delayed onset. Patients starting AED treatment, particularly with LEV, PER, and TPM, need long-term and comprehensive clinical monitoring with awareness of emergent adverse behavior.

## Figures and Tables

**Table 1:** Frequencies^∗^ of various psychiatric and behavioral adverse effects of levetiracetam, perampanel, and topiramate according to their European SPCs [24–26].

	Adverse effect	Comment
Levetiracetam	*Common*: Depression, **hostility/aggression**, anxiety, insomnia, nervousness/**irritability** *Uncommon*: Suicide attempt, suicidal ideation, psychotic disorder, abnormal behavior, hallucination, **anger**, confusion, panic attack, affect lability/mood swings, agitation *Rare*: Completed suicide, personality disorder, thinking abnormal	Higher prevalence in children and adolescents than in adults: agitation (3.4%), mood swings (2.1%), affect lability (1.7%), aggression (8.2%), abnormal behavior (5.6%)

Perampanel	*Common*: **Aggression**, **anger**, anxiety, confusion, **irritability** *Uncommon*: Suicidal ideation, suicide attempt	Aggression more frequently observed in adolescents than in adults

Topiramate	*Very common*: Depression *Common*: **Irritability**, bradyphrenia, insomnia, **expressive language disorder**, anxiety, confusion, disorientation, **aggression**, mood altered, agitation, mood swings, depressed mood, **anger**, abnormal behavior *Uncommon*: Suicidal ideation, suicide attempt, hallucination, psychotic disorder, hallucination auditory, hallucination visual, apathy, lack of spontaneous speech, sleep disorder, affect lability, libido decreased, restlessness, crying, dysphemia, euphoric mood, paranoia, perseveration, panic attack, tearfulness, reading disorder, initial insomnia, flat affect, thinking abnormal, loss of libido, listless, middle insomnia, distractibility, early morning awakening, panic reaction, elevated mood *Rare*: Mania, panic disorder, feeling of despair, hypomania	Irritability and expressive language among the most common adverse effects (>5%) Higher prevalence in children than in adults (>2 times): suicidal ideation, abnormal behavior, aggression

^∗^Very common: ≥1/10, common: ≥1/100 to <1/10, uncommon: ≥1/1000 to <1/100, rare: <1/1000.

**Table 2:** Studies reporting psychiatric and behavioral adverse reactions to levetiracetam.

Study	Study design	Study population	Main findings
Brodtkorb et al. 2004 [57]	Cohort study, *t* = 8.1 months	*n* = 184 adults (mean age: 34.7 years), of which 56 have intellectual disability	PBAR (aggression, irritability, mood swings, anxiety, restlessness, and psychotic symptoms) were among the most frequent adverse reactions. More frequent in patients with intellectual disability (23% vs. 10%).
Chen et al. 2017 [19]	Case-control, *t* = 1–15 years	*n* = 922 (2–18 years) with epilepsy; mono- or polytherapy	PBAR in 13.8%, leading to dose reduction or discontinuation in 11.2%. LEV with the highest frequency of PBAR (16.2%), leading to dose reduction or discontinuation in 6.7%.
Chen et al. 2017 [18]	Case-control, *t* = ≥12 months	*n* = 4085 adults (mean age 41 years) with epilepsy; mono- or polytherapy of which LEV: 1890	PBAR in 17.2%, leading to dose reduction or discontinuation in 13.8%. LEV with the highest frequency of these adverse reactions (22.1%), leading to dose reduction in 17.7%.
Chung et al. 2007 [50]	Cohort study, *t* = 2 years	*n* = 828 adults (mean age 38.5 years) (LEV: 196; LTG: 251; OXC: 97; TPM: 156; ZNS: 128)	Discontinuation due to PBAR in 19% using LEV (vs. 2–7% with LTG, OXC, TPM, and ZNS).
Ciesielski et al. 2006 [58]	Cohort study, *t* = 2 weeks	*n* = 20 (22–52 years) with epilepsy (LEV: 10, PGB: 10)	No difference in neuropsychological tests after short-term treatment with LEV or PGB.
Cramer et al. 2003 [32]	Review article, *t* = >2 years	Total *n* = 4179 adults (epilepsy, cognitive disorders, and anxiety) of which LEV: 2871, placebo: 1308	PBAR in 25.4% of 1393 patients using LEV (vs. 6.2% with placebo), including agitation (1.6% vs. 0.2%), emotional instability (3.0% vs. 0.2%), hostility (3.3% vs. 0.9%), and nervousness (7.3% vs. 1.8%). PBAR more common in epilepsy compared to non-epilepsy (cognition/anxiety) (*p* = 0.022).
de la Loge et al. 2010 [59]	RCT, *t* = 12 weeks	*n* = 98 (4–16 years), of which 64 used LEV as add-on and 34 used placebo	Significant difference in total problem score between LEV (worsened) vs. placebo (improved). Significant worsening of aggression (LEV vs. placebo; *p* = 0.013). Based on questionnaires.
Dinkelacker et al. 2003 [30]	Case series, *t* = 19 months	*n* = 33 adults with epilepsy	33 patients that experienced irritability or aggression (representing 3.5% of all patients treated with LEV, vs. <1% not on LEV). 24 patients: moderate or transient irritability, of which 10 had to reduce dose or discontinue. Nine (8 males) had severe aggressive symptoms; two of them required acute psychiatric intervention.
French et al. 2001 [60]	Review article, *t* = >3 years	*n* = 3347 adults (healthy subjects and patients with epilepsy or anxiety)	PBAR in 13% of 769 patients with epilepsy using LEV in placebo-controlled studies (placebo: 6%). 6% (placebo: 4.1%) of elderly and 5.1% (placebo: 5.5%) of patients with anxiety reported PBAR.
Guilfoyle et al. 2017 [61]	Case-control, *t* = 1 months	*n* = 335 children (mean age: 8.9 years) with newly diagnosed epilepsy, of which 37% started with LEV	Increased frequency of PBAR with any AED. LEV among those AEDs with the highest frequency.
Halma et al. 2014 [62]	Meta-analysis	*n* = 727 (1 month–18 years) with epilepsy using LEV as monotherapy or add-on. 13 studies in total	Three RCTs: hostility (7.3%), nervousness (6.1%), and aggression (4.9%). Significantly increased risk for these adverse reactions (relative risk: 2.2 vs. placebo; 95% KI: 1.4–3.4). Ten observational studies: worsened and improved behavior with LEV. Add-on therapy associated with irritability (4.7%), hyperexcitability (4.4%), and aggression (2.7%); monotherapy associated with general behavior problems (19%) and irritability (2.6%).
Helmstaedter et al. 2008 [63]	Interview-based, *t* = 2.3–5 years	*n* = 466, of which 288 used LEV (men age: 38 years), 135 relatives, and 43 controls (using different AEDs)	37% reported a negative behavior change, of which aggression was most frequent.
Kanemura et al. 2014 [64]	Cohort study, *t* = 12 months	*n* = 12 children (mean age: 10.3 years) with epilepsy and pervasive developmental disorder	Of eight patients with improved seizure control, six had >50% reduction in panic episodes or aggression.
Kang et al. 2013 [51]	Case-control, *t* = 29.3 months	*n* = 568 (mean age: 33 years) using LEV in mono- or polytherapy	Behavioral adverse reactions in up to 24%, of which irritability was most frequent.
Kowski et al. 2016 [65]	Case-control, *t* = 3 years	*n* = 841 patients with epilepsy (mean age: 44.7 years), of which 438 used monotherapy (different AEDs)	LEV with the highest frequency of anger, aggression, nervousness, and agitation
Labiner et al. 2009 [39]	RCT, *t* = 20 weeks	*n* = 268 patients with epilepsy (>16 years) of which 132 used LTG and 136 used LEV as add-on	Patients on LEV: worsened anger-aggression subscore, while patients on LTG improved each week.
Lee et al. 2011 [33]	Cohort study, *t* = 24 weeks	*n* = 71 patients with epilepsy (mean age: 35.4 years)	Improvement of anxiety symptoms with LEV, but five patients (6.5%) discontinued LEV due to PBAR (nervousness, irritability, anxiety, hostility, depression, suicidal ideation, and attempted suicide).
Mbizvo et al. 2014 [66]	Meta-analysis	*n* = 1861 children and adults, 11 studies in total	Agitation in 0.82% on LEV vs. 0.14% on placebo. Irritability in 0.46% vs. 0% on placebo.
Mula et al. 2003 [52]	Cohort study, *t* = 8.3 months	*n* = 517 patients (mean age: 35.6 years) using LEV as add-on	PBAR in 10%, of which aggression was most frequent (3.5%).
Mula et al. 2004 [31]	Cohort study, *t* = 8.3 months	*n* = 118 patients with epilepsy and learning disabilities (mean age: 30.6 years)	PBAR in 15 patients (12.7%). Aggression most common (9 patients; 7,6%). Two patients (1.7%) experienced agitation, anger, and hostility.
Mula et al. 2007 [67]	Case-control, *t* = 2 years	*n* = 108 patients with epilepsy (mean age: 37.9 years) using LEV and TPM (not simultaneously)	PBAR in 13%.
Mula et al. 2015 [68]	Case-control, interview	*n* = 163 (mean age: 42 years)	9.8% reported that aggressive behavior «always» was a problem.
Schiemann-Delgado et al. 2012 [69]	RCT, *t* = 48 weeks	*n* = 103 (4–16 years) of which 80 were from the de la Loge et al. (2010) study	No difference in score for behavior/aggression (LEV vs. placebo). Aggression occurred in 7.8%, irritability in 7.8% [sic], abnormal behavior in 3.9%.
Schoenberg et al. 2017 [70]	RCT, *t* = 10 weeks	*n* = 20 healthy elderly subjects, (mean age: 72.4 years) of which LEV: 9 and placebo: 11	LEV well tolerated regarding cognition, mood, and balance, but increased general tendency to feeling irritated (*p* = 0.029 vs. placebo).
Shukla et al. 2016 [71]	Case-control, *t* = 2.5 years	*n* = 445 patients with epilepsy (mean age: 21 years) using LEV (114), OXC (151), or VPA (134), of which 292 were included	PBAR in 43 patients (irritability, compulsive symptoms, aggression, psychosis). 23 (20.2%) used LEV. LEV discontinued in 10 patients (9%).
Tekgul et al. 2016 [49]	Case-control, *t* = ≥12 months	*n* = 351 (6 months–18 years: mean age: 9.9 years) using LEV in monotherapy	PBAR in 87%. Irritability (67%), hyperactivity (8%), and disturbed behavior (5%) were most common.
Weintraub et al. 2007 [27]	Case-control, *t* = 13 months	*n* = 1394 of which 521 patients (mean age: 43 years) used LEV	LEV with highest incidence (16%) of PBAR, leading to a discontinuation in 8%. Irritability in 9%, disturbed behavior in 3.5%.
White et al. 2003 [53]	Case-control, *t* = 25 months	*n* = 553 (mean age: 41.4 years)	7% discontinued LEV due to PBAR, mainly depression, and irritability. 1.8% were evaluated as a potential threat for themselves or others.
Wieshmann and Baker 2013 [72]	Case-control, interview	*n* = 459 (mean age: 41.6 years) of which 418 have epilepsy and 41 controls. 158 used LEV in monotherapy or add-on, 260 used other AEDs	49% of LEV users reported anger as a problem, vs. 3% using other AEDs, and 7% of controls.
Wieshmann and Baker 2017 [73]	Case-control, interview	*n* = 380 of which 329 (mean age: 39.8 years) have epilepsy using CBZ, VPA, LTG, or LEV in monotherapy, and 51 healthy controls	CNS-related adverse reactions more common with CBZ, VPA, LTG, and LEV vs. controls. Anger significantly more frequent with LEV (54% vs. 34% on CBZ, 33% on VPA, 31% on LTG, and 6% in controls).

RCT: randomized controlled trial, t: observation time; PBAR: psychiatric and/or behavioral adverse reactions; CBZ: carbamazepine; LEV: levetiracetam; LTG: lamotrigine; OXC: oxcarbazepine; PGB: pregabalin; TPM: topiramate; VPA: valproate; ZNS: zonisamide.

**Table 3:** Studies reporting psychiatric and behavioral adverse reactions to perampanel.

Study	Study design	Study population	Main findings
Biro et al. 2015 [35]	Case-control, *t* = 16 weeks–18 months	*n* = 58 (mean age: 10.5 years) treated with PER	Aggression in 8 patients (13.8%).
Chung et al. 2017 [43]	Case-control, *t* = 29–142 weeks	*n* = 1643 patients (≥12 years) with epilepsy using PER in monotherapy or with LEV and/or TPM	PER with increased risk of PBAR (incl. aggression, hostility, irritability, and anger). Occurrence of hostility and aggression independent of cotreatment with LEV or TPM.
Coyle et al. 2014 [74]	Case-control, *t* = 19 months	*n* = 47 patients with epilepsy (mean age: 31 years)	PBAR most common reason for discontinuation (aggression: *n* = 2; suicidal ideation *N* = 2; both combined: *n* = 1).
De Liso et al. 2016 [44]	Case-control, *t* = 7 months	*n* = 62 children/adolescents (mean age: 14.2 years) using PER as add-on	PBAR in 19 patients (30.6%), including irritability (*n* = 7; 11.3%) and aggression (*n* = 3; 4.8%).
Dolton and Choudry 2014 [75]	Case report, *t* = >6 months	1 patient (37 years) with epilepsy, Tourette's, moderately reduced cognitive function and demanding behavior	Add-on treatment with 8 mg PER improved seizure control but worsened aggressive behavior which resulted in institutionalization of the patient.
Ettinger et al. 2015 [34]	Review of safety in phase I, II, and III clinical studies	*n* = 9420 (12–>65 years) with epilepsy, Parkinson's, pain, MS, or migraine who received either PER or placebo	Higher incidence of aggression and hostility for PER vs. placebo in “narrow” and “broad” questionnaires (narrow: PER 3.0% vs. placebo 0.7%; broad: 11.8% vs. 5.7%), but not increased in non-epilepsy disorders.
French et al. 2015 [76]	RCT, *t* = 32–54 weeks	*n* = 162 patients (man age: 28.4 years) with generalized epilepsy, of which PER: 81 and placebo: 81	Irritability was the only individual adverse reaction with incidence ≥5% (PER: 11.1% vs. placebo 3.7%). Combined incidence of hostility and aggression: PER 18.5% vs. placebo 4.9%.
Huber and Schmid 2017 [37]	Case-control, *t* = 2 years	*n* = 26 patients (mean age: 30 years) with epilepsy and cognitive impairment of various degrees	PBAR in 50%, incl. irritability, aggression, increased sensitivity, and suicidal ideation/acts. This was also the main reason for discontinuation of PER.
Krauss et al. 2014 [77]	RCT, *t* = 1.5–>2 years	*n* = 1216 patients (≥12 years) with epilepsy, using 1–3 AEDs and PER as add-on	Irritability in 11.5% and aggression in 5.1%, leading to discontinuation of PER in 1.3% and 0.4%, respectively. 3.9% had ≥1 serious PBAR, of which 0.2% agitation, 0.2% abnormal behavior, and 1% aggression.
Lagae et al. 2016 [78]	RCT, *t* = 20 weeks	*n* = 133 (12–17 years) with epilepsy (PER: 85 and placebo: 48)	No difference in total score (behavior and competence) between PER and placebo, but aggression and hostility in 15 patients (17.6%) on PER vs. 2 (4.2%) on placebo.
Rosenfeld et al. 2015 [45]	RCT, *t* = 25–29 weeks	*n* = 143 (12–17 years) with epilepsy of which PER: 98 and placebo: 45	Aggression in 8.2% (vs. 0% on placebo). Aggression was one of the most common reasons (6.6%) for dose changes or discontinuation of PER
Rugg-Gunn 2014 [46]	Review article, *t* = ≥19 weeks	*n* = 1450 patients of which 1008 on PER and 442 on placebo	Higher frequency of PBAR with PER, particularly irritability and aggression. Frequency of serious PBAR reported as low, but 3 cases of aggression and 1 of suicidal ideation.
Snoeijen-Schouwenaars et al. 2017 [36]	Case-control, *t* = 12 months	*n* = 62 patients (mean age: 27.4 years) with epilepsy and intellectual disability	Behavioral adverse reactions in 40.3%. Most common: aggression, agitation, disturbing behavior, and mood symptoms.
Steinhoff et al. 2013 [47]	RCT, *t* = 25 weeks	*n* = 1478 of which PER: 1038 (mean age: 34.8 years) or placebo: 442 (mean age: 34.3 years)	Irritability in 11.8% on 12 mg PER (vs. 2.9% on placebo and 3.9–6.7% on 2–8 mg PER). Aggression in 3% on 12 mg PER (vs. 1% on placebo, 1% on 4 mg PER, and 2% on 8 mg PER). Hostility or aggression in 5% (4 mg), 12% (8 mg), and 20% (12 mg) on PER, vs. 6% on placebo
Steinhoff et al. 2014 [79]	Cohort study, *t* = ≥6 months	*n* = 281 patients (≥12 years) with focal seizures	Aggression in 2.8%, irritability in 2.1%.
Wehner et al. 2017 [80]	Cohort study, *t* = 38–42 months	*n* = 391 patients (≥17 years) using PER as add-on	Negative effect on mental health in 137 patients (36%), incl. worsened mood, increased irritability and demanding behavior
Zaccara et al. 2013 [48]	Meta-analysis	*n* = 3947 patients with epilepsy or Parkinson's, of which 2627 used PER in a total of 9 RCTs	Irritability and aggression with a PER dose of 12 mg/day. Overall tolerability was better in epilepsy compared to Parkinson's, but patients with Parkinson's were older.

RCT: randomized controlled trial, t: observation time; PBAR: psychiatric and/or behavioral adverse reactions; LEV: levetiracetam; PER: perampanel; TPM: topiramate.

**Table 4:** Studies reporting psychiatric and behavioral adverse reactions to topiramate.

Study	Study design	Study population	Main findings
Chen et al. 2017 [18]	Case-control, *t* = ≥1 years	*n* = 4085 adults (mean age: 41 years) with epilepsy on ≥1 AED, of which TPM: 639	PBAR in 17.2%, leading to dose reduction or discontinuation in 13.8% (all patients) and 6.3% (TPM users).
Chung et al. 2007 [50]	Case-control, *t* = 2 years	*n* = 828 adults (mean age 38.5 years) on different AEDs (LEV: 196, LTG: 251, OXC: 97, TPM: 156, ZNS: 128)	TPM with the highest rate of discontinuation (55.8%), but only few due to PBAR (5 of 156 patients).
Endoh et al. 2012 [54]	Case-control, *t* = 17.6 months	*n* = 58 children with epileptic spasms, of which 33 used TPM	5 of 33 patients (15.2%) developed irritability.
Grosso et al. 2005 [81]	Cohort study, *t* = 11 months	*n* = 59 children < 2 years (mean age: 13 months) on TPM	Irritability is one of the most common adverse reactions.
Kanner et al. 2003 [82]	Cohort study, *t* = 10.5 months	*n* = 596 patients (mean age: 36.1 years) with epilepsy using TPM as monotherapy or add-on	PBAR in 12.6%, incl. aggression (10.7%), irritability (5.7%), and depression (5%). TPM discontinued in 27% with these adverse reactions.
Lee et al. 2011 [55]	Cohort study, *t* = 17.2 weeks	*n* = 28 children (2-18 months) with infantile spasms using TPM	Irritability in 4 patients (14.3%; most common adverse reaction).
Mula et al. 2003 [38]	Cohort study, *t* = ≥6 months	*n* = 431 patients (mean age 35.8 years) with epilepsy using TPM	PBAR in 24% (aggression: 5.6%).
Mula and Trimble 2003 [56]	Cohort study, *t* = ≥6 months	*n* = 103 patients on TPM	Mood symptoms in almost half of patients. Aggression is the second most common (23%), resolved after dose reduction or discontinuation of TPM.
Mula et al. 2007 [67]	Case-control, *t* = 2 years	*n* = 108 patients with epilepsy, treated with LEV and TPM (consecutively)	PBAR in 30%
Reith et al. 2003 [83]	Case-control, *t* = 309 days	*n* = 159< 18 years (mean age: 8.1 years) with epilepsy using TPM; follow-up of *n* = 127 of these	Aggression or psychosis treatment-limiting in 10 of 127 patients (7.9%).
Weintraub et al. 2007 [27]	Case-control, *t* = 13 months	*n* = 1394 of which 112 patients (mean age: 41 years) used TPM	PBAR in 6.3% on TPM, which was lower than the mean frequency of all AEDs (8.4%)

RCT: randomized controlled trial, t: observation time; PBAR: psychiatric and/or behavioral adverse reactions; LEV: levetiracetam; LTG: lamotrigine; OXC: oxcarbazepine; TPM: topiramate, ZNS: zonisamide.

## References

[1] Ngugi A. K., Bottomley C., Kleinschmidt I., Sander J. W., Newton C. R. (2010). Estimation of the burden of active and life-time epilepsy: a meta-analytic approach. *Epilepsia*.

[2] Hirtz D., Thurman D. J., Gwinn-Hardy K., Mohamed M., Chaudhuri A. R., Zalutsky R. (2007). How common are the “common” neurologic disorders?. *Neurology*.

[3] Brodie M. J., Bamagous G., Kwan P. (2009). Improved outcomes in newly diagnosed epilepsy. *Epilepsia*.

[4] Brodie M. J., Besag F., Ettinger A. B. (2016). Epilepsy, antiepileptic drugs, and aggression: an evidence-based review. *Pharmacological Reviews*.

[5] Andres E., Kerling F., Hamer H., Winterholler M. (2018). Behavioural changes in patients with intellectual disability treated with brivaracetam. *Acta Neurologica Scandinavica*.

[6] Steinig I., von Podewils F., Moddel G. (2017). Postmarketing experience with brivaracetam in the treatment of epilepsies: a multicenter cohort study from Germany. *Epilepsia*.

[7] Lin J. J., Mula M., Hermann B. P. (2012). Uncovering the neurobehavioural comorbidities of epilepsy over the lifespan. *The Lancet*.

[8] Alper K. R., Barry J. J., Balabanov A. J. (2002). Treatment of psychosis, aggression, and irritability in patients with epilepsy. *Epilepsy & Behavior*.

[9] Calles J. L. (2016). Aggressive behaviors. *Journal of Alternative Medicine Research*.

[10] Crocker A. G., Mercier C., Lachapelle Y., Brunet A., Morin D., Roy M. E. (2006). Prevalence and types of aggressive behaviour among adults with intellectual disabilities. *Journal of Intellectual Disability Research*.

[11] Munshi K. R., Oken T., Guild D. J. (2010). The use of antiepileptic drugs (AEDs) for the treatment of pediatric aggression and mood disorders. *Pharmaceuticals*.

[12] Comai S., Tau M., Gobbi G. (2012). The psychopharmacology of aggressive behavior: a translational approach: part 1: neurobiology. *Journal of Clinical Psychopharmacology*.

[13] Comai S., Tau M., Pavlovic Z., Gobbi G. (2012). The psychopharmacology of aggressive behavior: a translational approach. *Journal of Clinical Psychopharmacology*.

[14] Nelson R. J., Chiavegatto S. (2001). Molecular basis of aggression. *Trends in Neurosciences*.

[15] Giussani G., Bianchi E., Canelli V. (2017). Antiepileptic drug discontinuation by people with epilepsy in the general population. *Epilepsia*.

[16] Marson A. G., Al-Kharusi A. M., Alwaidh M. (2007). The SANAD study of effectiveness of carbamazepine, gabapentin, lamotrigine, oxcarbazepine, or topiramate for treatment of partial epilepsy: an unblinded randomised controlled trial. *The Lancet*.

[17] Perucca P., Gilliam F. G. (2012). Adverse effects of antiepileptic drugs. *Lancet Neurology*.

[18] Chen B., Choi H., Hirsch L. J. (2017). Psychiatric and behavioral side effects of antiepileptic drugs in adults with epilepsy. *Epilepsy & Behavior*.

[19] Chen B., Detyniecki K., Choi H. (2017). Psychiatric and behavioral side effects of anti-epileptic drugs in adolescents and children with epilepsy. *European Journal of Paediatric Neurology*.

[20] Besag F. M. C. (2017). Risk factors for psychiatric and behavioural adverse events associated with antiepileptic drugs in adolescents and children. *European Journal of Paediatric Neurology*.

[21] Stephen L. J., Wishart A., Brodie M. J. (2017). Psychiatric side effects and antiepileptic drugs: observations from prospective audits. *Epilepsy & Behavior*.

[22] Ortega G., Abraira L., Marti G. (2018). Anger assessment in patients treated with brivaracetam. *Clinical Neuropharmacology*.

[23] Toledo M., Whitesides J., Schiemann J. (2016). Safety, tolerability, and seizure control during long-term treatment with adjunctive brivaracetam for partial-onset seizures. *Epilepsia*.

[24] Keppra European SPC EMA. http://www.ema.europa.eu/docs/no_NO/document_library/EPAR_-_Product_Information/human/000277/WC500041334.pdf.

[25] Fycompa European SPC EMA. http://www.ema.europa.eu/docs/no_NO/document_library/EPAR_-_Product_Information/human/002434/WC500130815.pdf.

[26] Topamax European SPC EMA. https://www.legemiddelsok.no/_layouts/15/Preparatomtaler/Spc/1995-00790.pdf.

[27] Weintraub D., Buchsbaum R., Resor S. R., Hirsch L. J. (2007). Psychiatric and behavioral side effects of the newer antiepileptic drugs in adults with epilepsy. *Epilepsy & Behavior*.

[28] Eddy C. M., Rickards H. E., Cavanna A. E. (2012). Behavioral adverse effects of antiepileptic drugs in epilepsy. *Journal of Clinical Psychopharmacology*.

[29] Briviact European SPC EMA. http://www.ema.europa.eu/docs/en_GB/document_library/EPAR_-_Product_Information/human/003898/WC500200206.pdf.

[30] Dinkelacker V., Dietl T., Widman G., Lengler U., Elger C. E. (2003). Aggressive behavior of epilepsy patients in the course of levetiracetam add-on therapy: report of 33 mild to severe cases. *Epilepsy & Behavior*.

[31] Mula M., Trimble M. R., Sander J. W. (2004). Psychiatric adverse events in patients with epilepsy and learning disabilities taking levetiracetam. *Seizure*.

[32] Cramer J. A., De Rue K., Devinsky O., Edrich P., Trimble M. R. (2003). A systematic review of the behavioral effects of levetiracetam in adults with epilepsy, cognitive disorders, or an anxiety disorder during clinical trials. *Epilepsy & Behavior*.

[33] Lee J. J., Song H. S., Hwang Y. H., Lee H. W., Suh C. K., Park S. P. (2011). Psychiatric symptoms and quality of life in patients with drug-refractory epilepsy receiving adjunctive levetiracetam therapy. *Journal of Clinical Neurology*.

[34] Ettinger A. B., LoPresti A., Yang H. (2015). Psychiatric and behavioral adverse events in randomized clinical studies of the noncompetitive AMPA receptor antagonist perampanel. *Epilepsia*.

[35] Biro A., Stephani U., Tarallo T. (2015). Effectiveness and tolerability of perampanel in children and adolescents with refractory epilepsies: first experiences. *Neuropediatrics*.

[36] Snoeijen-Schouwenaars F. M., van Ool J. S., Tan I. Y., Schelhaas H. J., Majoie M. H. (2017). Evaluation of perampanel in patients with intellectual disability and epilepsy. *Epilepsy & Behavior*.

[37] Huber B., Schmid G. (2017). A two-year retrospective evaluation of perampanel in patients with highly drug-resistant epilepsy and cognitive impairment. *Epilepsy & Behavior*.

[38] Mula M., Trimble M. R., Lhatoo S. D., Sander J. W. (2003). Topiramate and psychiatric adverse events in patients with epilepsy. *Epilepsia*.

[39] Labiner D. M., Ettinger A. B., Fakhoury T. A. (2009). Effects of lamotrigine compared with levetiracetam on anger, hostility, and total mood in patients with partial epilepsy. *Epilepsia*.

[40] Moavero R., Santarone M. E., Galasso C., Curatolo P. (2017). Cognitive and behavioral effects of new antiepileptic drugs in pediatric epilepsy. *Brain & Development*.

[41] Nadkarni S., Devinsky O. (2005). Psychotropic effects of antiepileptic drugs. *Epilepsy Currents*.

[42] Schmitz B. (2006). Effects of antiepileptic drugs on mood and behavior. *Epilepsia*.

[43] Chung S., Williams B., Dobrinsky C., Patten A., Yang H., Laurenza A. (2017). Perampanel with concomitant levetiracetam and topiramate: post hoc analysis of adverse events related to hostility and aggression. *Epilepsy & Behavior*.

[44] De Liso P., Vigevano F., Specchio N. (2016). Effectiveness and tolerability of perampanel in children and adolescents with refractory epilepsies—an Italian observational multicenter study. *Epilepsy Research*.

[45] Rosenfeld W., Conry J., Lagae L. (2015). Efficacy and safety of perampanel in adolescent patients with drug-resistant partial seizures in three double-blind, placebo-controlled, phase III randomized clinical studies and a combined extension study. *European Journal of Paediatric Neurology*.

[46] Rugg-Gunn F. (2014). Adverse effects and safety profile of perampanel: a review of pooled data. *Epilepsia*.

[47] Steinhoff B. J., Ben-Menachem E., Ryvlin P. (2013). Efficacy and safety of adjunctive perampanel for the treatment of refractory partial seizures: a pooled analysis of three phase III studies. *Epilepsia*.

[48] Zaccara G., Giovannelli F., Cincotta M., Verrotti A., Grillo E. (2013). The adverse event profile of perampanel: meta-analysis of randomized controlled trials. *European Journal of Neurology*.

[49] Tekgul H., Gencpinar P., Cavusoglu D., Dundar N. O. (2016). The efficacy, tolerability and safety of levetiracetam therapy in a pediatric population. *Seizure*.

[50] Chung S., Wang N., Hank N. (2007). Comparative retention rates and long-term tolerability of new antiepileptic drugs. *Seizure*.

[51] Kang B. S., Moon H. J., Kim Y. S. (2013). The long-term efficacy and safety of levetiracetam in a tertiary epilepsy centre. *Epileptic Disorders*.

[52] Mula M., Trimble M. R., Yuen A., Liu R. S., Sander J. W. (2003). Psychiatric adverse events during levetiracetam therapy. *Neurology*.

[53] White J. R., Walczak T. S., Leppik I. E. (2003). Discontinuation of levetiracetam because of behavioral side effects: a case-control study. *Neurology*.

[54] Endoh F., Kobayashi K., Hayashi Y., Shibata T., Yoshinaga H., Ohtsuka Y. (2012). Efficacy of topiramate for intractable childhood generalized epilepsy with epileptic spasms: with special reference to electroencephalographic changes. *Seizure*.

[55] Lee G. M., Lee K. S., Lee E. H., Chung S. (2011). Short term outcomes of topiramate monotherapy as a first-line treatment in newly diagnosed West syndrome. *Korean Journal of Pediatrics*.

[56] Mula M., Trimble M. R. (2003). The importance of being seizure free: topiramate and psychopathology in epilepsy. *Epilepsy & Behavior*.

[57] Brodtkorb E., Klees T. M., Nakken K. O., Lossius R., Johannessen S. I. (2004). Levetiracetam in adult patients with and without learning disability: focus on behavioral adverse effects. *Epilepsy & Behavior*.

[58] Ciesielski A. S., Samson S., Steinhoff B. J. (2006). Neuropsychological and psychiatric impact of add-on titration of pregabalin versus levetiracetam: a comparative short-term study. *Epilepsy & Behavior*.

[59] de la Loge C., Hunter S. J., Schiemann J., Yang H. (2010). Assessment of behavioral and emotional functioning using standardized instruments in children and adolescents with partial-onset seizures treated with adjunctive levetiracetam in a randomized, placebo-controlled trial. *Epilepsy & Behavior*.

[60] French J., Edrich P., Cramer J. A. (2001). A systematic review of the safety profile of levetiracetam: a new antiepileptic drug. *Epilepsy Research*.

[61] Guilfoyle S. M., Follansbee-Junger K., Smith A. W. (2017). Antiepileptic drug behavioral side effects and baseline hyperactivity in children and adolescents with new onset epilepsy. *Epilepsia*.

[62] Halma E., de Louw A. J., Klinkenberg S., Aldenkamp A. P., DM I. J., Majoie M. (2014). Behavioral side-effects of levetiracetam in children with epilepsy: a systematic review. *Seizure*.

[63] Helmstaedter C., Fritz N. E., Kockelmann E., Kosanetzky N., Elger C. E. (2008). Positive and negative psychotropic effects of levetiracetam. *Epilepsy & Behavior*.

[64] Kanemura H., Sano F., Ohyama T., Sugita K., Aihara M. (2014). Effect of levetiracetam on behavioral problems in pervasive developmental disorder children with epilepsy. *European Journal of Paediatric Neurology*.

[65] Kowski A. B., Weissinger F., Gaus V., Fidzinski P., Losch F., Holtkamp M. (2016). Specific adverse effects of antiepileptic drugs — a true-to-life monotherapy study. *Epilepsy & Behavior*.

[66] Mbizvo G. K., Dixon P., Hutton J. L., Marson A. G. (2014). The adverse effects profile of levetiracetam in epilepsy: a more detailed look. *International Journal of Neuroscience*.

[67] Mula M., Trimble M. R., Sander J. W. (2007). Are psychiatric adverse events of antiepileptic drugs a unique entity? A study on topiramate and levetiracetam. *Epilepsia*.

[68] Mula M., Agrawal N., Mustafa Z. (2015). Self-reported aggressiveness during treatment with levetiracetam correlates with depression. *Epilepsy & Behavior*.

[69] Schiemann-Delgado J., Yang H., Loge Cde L. (2012). A long-term open-label extension study assessing cognition and behavior, tolerability, safety, and efficacy of adjunctive levetiracetam in children aged 4 to 16 years with partial-onset seizures. *Journal of Child Neurology*.

[70] Schoenberg M. R., Rum R. S., Osborn K. E., Werz M. A. (2017). A randomized, double-blind, placebo-controlled crossover study of the effects of levetiracetam on cognition, mood, and balance in healthy older adults. *Epilepsia*.

[71] Shukla G., Gupta A., Agarwal P., Poornima S. (2016). Behavioral effects and somnolence due to levetiracetam versus oxcarbazepine - a retrospective comparison study of North Indian patients with refractory epilepsy. *Epilepsy & Behavior*.

[72] Wieshmann U. C., Baker G. A. (2013). Self-reported feelings of anger and aggression towards others in patients on levetiracetam: data from the UK antiepileptic drug register. *BMJ Open*.

[73] Wieshmann U. C., Baker G. (2017). Efficacy and tolerability of anti-epileptic drugs-an internet study. *Acta Neurologica Scandinavica*.

[74] Coyle H., Clough P., Cooper P., Mohanraj R. (2014). Clinical experience with perampanel: focus on psychiatric adverse effects. *Epilepsy & Behavior*.

[75] Dolton E., Choudry A. (2014). Perampanel and challenging behaviour in intellectual disability and epilepsy: a management dilemma. *Case Reports in Psychiatry*.

[76] French J. A., Krauss G. L., Wechsler R. T. (2015). Perampanel for tonic-clonic seizures in idiopathic generalized epilepsy a randomized trial. *Neurology*.

[77] Krauss G. L., Perucca E., Ben-Menachem E. (2014). Long-term safety of perampanel and seizure outcomes in refractory partial-onset seizures and secondarily generalized seizures: results from phase III extension study 307. *Epilepsia*.

[78] Lagae L., Villanueva V., Meador K. J. (2016). Adjunctive perampanel in adolescents with inadequately controlled partial-onset seizures: a randomized study evaluating behavior, efficacy, and safety. *Epilepsia*.

[79] Steinhoff B. J., Hamer H., Trinka E. (2014). A multicenter survey of clinical experiences with perampanel in real life in Germany and Austria. *Epilepsy Research*.

[80] Wehner T., Mannan S., Turaga S. (2017). Retention of perampanel in adults with pharmacoresistant epilepsy at a single tertiary care center. *Epilepsy & Behavior*.

[81] Grosso S., Galimberti D., Farnetani M. A. (2005). Efficacy and safety of topiramate in infants according to epilepsy syndromes. *Seizure*.

[82] Kanner A. M., Wuu J., Faught E., Tatum W. O., Fix A., French J. A. (2003). A past psychiatric history may be a risk factor for topiramate-related psychiatric and cognitive adverse events. *Epilepsy & Behavior*.

[83] Reith D., Burke C., Appleton D. B., Wallace G., Pelekanos J. (2003). Tolerability of topiramate in children and adolescents. *Journal of Paediatrics and Child Health*.

[84] Cortes-Altamirano J. L., Olmos-Hernandez A., Bonilla-Jaime H., Bandala C., Gonzalez-Maciel A., Alfaro-Rodriguez A. (2016). Levetiracetam as an antiepileptic, neuroprotective, and hyperalgesic drug. *Neurology India*.

[85] Lyseng-Williamson K. A. (2011). Spotlight on levetiracetam in epilepsy. *CNS Drugs*.

[86] Rogawski M. A. (2006). Diverse mechanisms of antiepileptic drugs in the development pipeline. *Epilepsy Research*.

[87] Carunchio I., Pieri M., Ciotti M. T., Albo F., Zona C. (2007). Modulation of AMPA receptors in cultured cortical neurons induced by the antiepileptic drug levetiracetam. *Epilepsia*.

[88] Doelken M. T., Hammen T., Bogner W. (2010). Alterations of intracerebral *γ*-aminobutyric acid (GABA) levels by titration with levetiracetam in patients with focal epilepsies. *Epilepsia*.

[89] Luz Adriana P. M., Blanca Alcira R. M., Itzel Jatziri C. G. (2018). Effect of levetiracetam on extracellular amino acid levels in the dorsal hippocampus of rats with temporal lobe epilepsy. *Epilepsy Research*.

[90] Rigo J. M., Hans G., Nguyen L. (2002). The anti-epileptic drug levetiracetam reverses the inhibition by negative allosteric modulators of neuronal GABA- and glycine-gated currents. *British Journal of Pharmacology*.

[91] Ueda Y., Doi T., Nagatomo K., Tokumaru J., Takaki M., Willmore L. J. (2007). Effect of levetiracetam on molecular regulation of hippocampal glutamate and GABA transporters in rats with chronic seizures induced by amygdalar FeCl3 injection. *Brain Research*.

[92] Wakita M., Kotani N., Kogure K., Akaike N. (2014). Inhibition of excitatory synaptic transmission in hippocampal neurons by levetiracetam involves Zn^2+^-dependent GABA type a receptor–mediated presynaptic modulation. *The Journal of Pharmacology and Experimental Therapeutics*.

[93] Lynch B. A., Lambeng N., Nocka K. (2004). The synaptic vesicle protein SV2A is the binding site for the antiepileptic drug levetiracetam. *Proceedings of the National Academy of Sciences of the United States of America*.

[94] Lee C. Y., Chen C. C., Liou H. H. (2009). Levetiracetam inhibits glutamate transmission through presynaptic P/Q-type calcium channels on the granule cells of the dentate gyrus. *British Journal of Pharmacology*.

[95] Steinhoff B. J., Bacher M., Bucurenciu I. (2017). Real-life experience with brivaracetam in 101 patients with difficult-to-treat epilepsy—a monocenter survey. *Seizure*.

[96] Yates S. L., Fakhoury T., Liang W., Eckhardt K., Borghs S., D'Souza J. (2015). An open-label, prospective, exploratory study of patients with epilepsy switching from levetiracetam to brivaracetam. *Epilepsy & Behavior*.

[97] Niespodziany I., Rigo J. M., Moonen G., Matagne A., Klitgaard H., Wolff C. (2017). Brivaracetam does not modulate ionotropic channels activated by glutamate, *γ*‐aminobutyric acid, and glycine in hippocampal neurons. *Epilepsia*.

[98] Winblad B. (2005). Piracetam: a review of pharmacological properties and clinical uses. *CNS Drug Reviews*.

[99] Ahmed A. H., Oswald R. E. (2010). Piracetam defines a new binding site for allosteric modulators of alpha-amino-3-hydroxy-5-methyl-4-isoxazole-propionic acid (AMPA) receptors. *Journal of Medicinal Chemistry*.

[100] de Almeida R. M., Ferrari P. F., Parmigiani S., Miczek K. A. (2005). Escalated aggressive behavior: dopamine, serotonin and GABA. *European Journal of Pharmacology*.

[101] Coccaro E. F., Fanning J. R., Phan K. L., Lee R. (2015). Serotonin and impulsive aggression. *CNS Spectrums*.

[102] Takahashi A., Quadros I. M., de Almeida R. M., Miczek K. A. (2011). Brain serotonin receptors and transporters: initiation vs. termination of escalated aggression. *Psychopharmacology*.

[103] Rudzinski L. A., Velez-Ruiz N. J., Gedzelman E. R., Mauricio E. A., Shih J. J., Karakis I. (2016). New antiepileptic drugs: focus on ezogabine, clobazam, and perampanel. *Journal of Investigative Medicine*.

[104] Coccaro E. F., Lee R., Vezina P. (2013). Cerebrospinal fluid glutamate concentration correlates with impulsive aggression in human subjects. *Journal of Psychiatric Research*.

[105] Vekovischeva O. Y., Aitta-Aho T., Echenko O. (2004). Reduced aggression in AMPA-type glutamate receptor GluR-A subunit-deficient mice. *Genes, brain, and behavior*.

[106] Vekovischeva O. Y., Aitta-aho T., Verbitskaya E., Sandnabba K., Korpi E. R. (2007). Acute effects of AMPA-type glutamate receptor antagonists on intermale social behavior in two mouse lines bidirectionally selected for offensive aggression. *Pharmacology, Biochemistry, and Behavior*.

[107] Araki R., Ago Y., Hasebe S. (2014). Involvement of prefrontal AMPA receptors in encounter stimulation-induced hyperactivity in isolation-reared mice. *International Journal of Neuropsychopharmacology*.

[108] Belozertseva I. V., Bespalov A. Y. (1999). Effects of NMDA receptor channel blockade on aggression in isolated male mice. *Aggressive Behavior*.

[109] Spritzer S. D., Bravo T. P., Drazkowski J. F. (2016). Topiramate for treatment in patients with migraine and epilepsy. *Headache*.

[110] Shank R. P., Gardocki J. F., Streeter A. J., Maryanoff B. E. (2000). An overview of the preclinical aspects of topiramate: pharmacology, pharmacokinetics, and mechanism of action. *Epilepsia*.

[111] Kudin A. P., Debska-Vielhaber G., Vielhaber S., Elger C. E., Kunz W. S. (2004). The mechanism of neuroprotection by topiramate in an animal model of epilepsy. *Epilepsia*.

[112] Carney P. W., Jackson G. D. (2014). Insights into the mechanisms of absence seizure generation provided by EEG with functional MRI. *Frontiers in Neurology*.

[113] Baykan B., Wolf P. (2017). Juvenile myoclonic epilepsy as a spectrum disorder: a focused review. *Seizure*.

[114] Syvertsen M. R., Thuve S., Stordrange B. S., Brodtkorb E. (2014). Clinical heterogeneity of juvenile myoclonic epilepsy: follow-up after an interval of more than 20 years. *Seizure*.

[115] Mula M., Monaco F. (2009). Antiepileptic drugs and psychopathology of epilepsy: an update. *Epileptic Disorders*.

[116] Marsh L., Krauss G. L. (2000). Aggression and violence in patients with epilepsy. *Epilepsy & Behavior*.

[117] Mula M. (2016). The interictal dysphoric disorder of epilepsy: legend or reality?. *Epilepsy & Behavior*.

[118] Berg A. T., Altalib H. H., Devinsky O. (2017). Psychiatric and behavioral comorbidities in epilepsy: a critical reappraisal. *Epilepsia*.

[119] Devinsky O. (2008). Postictal psychosis: common, dangerous, and treatable. *Epilepsy Currents*.

[120] Eisenschenk S., Krop H., Devinsky O. (2014). Homicide during postictal psychosis. *Epilepsy & Behavior Case Reports*.

[121] Mullen S. A., Carvill G. L., Bellows S. (2013). Copy number variants are frequent in genetic generalized epilepsy with intellectual disability. *Neurology*.

[122] Johnson M. R., Shorvon S. D. (2011). Heredity in epilepsy: neurodevelopment, comorbidity, and the neurological trait. *Epilepsy & Behavior*.

[123] Torres F., Barbosa M., Maciel P. (2016). Recurrent copy number variations as risk factors for neurodevelopmental disorders: critical overview and analysis of clinical implications. *Journal of Medical Genetics*.

[124] Vlaskamp D. R. M., Callenbach P. M. C., Rump P. (2017). Copy number variation in a hospital-based cohort of children with epilepsy. *Epilepsia Open*.

[125] Helmstaedter C., Mihov Y., Toliat M. R. (2013). Genetic variation in dopaminergic activity is associated with the risk for psychiatric side effects of levetiracetam. *Epilepsia*.

[126] Brodtkorb E., Shorvon S., Perucca E., Engel J. (2016). Management of epilepsy in people with intellectual disabilities. *The Treatment of Epilepsi*.

[127] Besag F. M. (2004). Behavioural effects of the newer antiepileptic drugs: an update. *Expert Opinion on Drug Safety*.

[128] Loring D. W., Marino S., Meador K. J. (2007). Neuropsychological and behavioral effects of antiepilepsy drugs. *Neuropsychology Review*.

[129] Witt J. A., Elger C. E., Helmstaedter C. (2013). Impaired verbal fluency under topiramate--evidence for synergistic negative effects of epilepsy, topiramate, and polytherapy. *European Journal of Neurology*.

[130] Birger M., Swartz M., Cohen D., Alesh Y., Grishpan C., Kotelr M. (2003). Aggression: the testosterone-serotonin link. *Israel Medical Association Journal*.

[131] Carre J. M., Geniole S. N., Ortiz T. L., Bird B. M., Videto A., Bonin P. L. (2017). Exogenous testosterone rapidly increases aggressive behavior in dominant and impulsive men. *Biological Psychiatry*.

[132] Swann A. C. (2003). Neuroreceptor mechanisms of aggression and its treatment. *Journal of Clinical Psychiatry*.

[133] Clark A. S., Henderson L. P. (2003). Behavioral and physiological responses to anabolic-androgenic steroids. *Neuroscience and Biobehavioral Reviews*.

[134] Rodin E. A. (1973). Psychomotor epilepsy and aggressive behavior. *Archives of General Psychiatry*.

[135] Pandya N. S., Vrbancic M., Ladino L. D., Tellez-Zenteno J. F. (2013). Epilepsy and homicide. *Neuropsychiatric Disease and Treatment*.

[136] Piazzini A., Turner K., Edefonti V. (2011). A new Italian instrument for the assessment of irritability in patients with epilepsy. *Epilepsy & Behavior*.

[137] Reimers A. (2014). New antiepileptic drugs and women. *Seizure*.

[138] Svalheim S., Sveberg L., Mochol M., Tauboll E. (2015). Interactions between antiepileptic drugs and hormones. *Seizure*.

[139] Maguire J., Salpekar J. A. (2013). Stress, seizures, and hypothalamic–pituitary–adrenal axis targets for the treatment of epilepsy. *Epilepsy & Behavior*.

[140] Summers C. H., Winberg S. (2006). Interactions between the neural regulation of stress and aggression. *The Journal of Experimental Biology*.

[141] Berger S. L., Kouzarides T., Shiekhattar R., Shilatifard A. (2009). An operational definition of epigenetics. *Genes & Development*.

[142] Elvir L., Duclot F., Wang Z., Kabbaj M. (2017). Epigenetic regulation of motivated behaviors by histone deacetylase inhibitors. *Neuroscience and Biobehavioral Reviews*.

[143] Eyal S., Yagen B., Sobol E., Altschuler Y., Shmuel M., Bialer M. (2004). The activity of antiepileptic drugs as histone deacetylase inhibitors. *Epilepsia*.

[144] Landolt H. (1953). Some clinical electroencephalographic correlations in epileptic psychoses (twilight states). *Electroencephalography and Clinical Neurophysiology*.

[145] Glauser T. A. (2004). Effects of antiepileptic medications on psychiatric and behavioral comorbidities in children and adolescents with epilepsy. *Epilepsy & Behavior*.

[146] Kawakami Y., Itoh Y. (2017). Forced normalization: antagonism between epilepsy and psychosis. *Pediatric Neurology*.

[147] Loganathan M. A., Enja M., Lippmann S. (2015). FORCED NORMALIZATION: epilepsy and psychosis interaction. *Innovations in Clinical Neuroscience*.

[148] Baran B., Bitter I., Ungvari G. S., Gazdag G. (2012). The birth of convulsive therapy revisited: a reappraisal of Laszlo Meduna’s first cohort of patients. *Journal of Affective Disorders*.

[149] Topkan A., Bilen S., Titiz A. P., Eruyar E., Ak F. (2016). Forced normalization: an overlooked entity in epileptic patients. *Asian Journal of Psychiatry*.

[150] Longo L. P., Johnson B. (2000). Addiction: Part I. Benzodiazepines--side effects, abuse risk and alternatives. *American Family Physician*.

[151] Sharma T., Guski L. S., Freund N., Gotzsche P. C. (2016). Suicidality and aggression during antidepressant treatment: systematic review and meta-analyses based on clinical study reports. *BMJ*.

[152] Stuckelman Z. D., Mulqueen J. M., Ferracioli-Oda E. (2017). Risk of irritability with psychostimulant treatment in children with ADHD: a meta-analysis. *The Journal of Clinical Psychiatry*.

[153] Varghese B. S., Rajeev A., Norrish M., Khusaiby S. B. (2010). Topiramate for anger control: a systematic review. *Indian Journal of Pharmacology*.

[154] de Vries T. W., van Hunsel F. (2016). Adverse drug reactions of systemic antihistamines in children in the Netherlands. *Archives of Disease in Childhood*.

[155] Golomb B. A., Kane T., Dimsdale J. E. (2004). Severe irritability associated with statin cholesterol-lowering drugs. *QJM*.

[156] Morrison T. R., Sikes R. W., Melloni R. H. (2016). Anabolic steroids alter the physiological activity of aggression circuits in the lateral anterior hypothalamus. *Neuroscience*.

[157] Salas-Ramirez K. Y., Montalto P. R., Sisk C. L. (2010). Anabolic steroids have long-lasting effects on male social behaviors. *Behavioural Brain Research*.

[158] Carrillo M., Ricci L. A., Melloni R. H. (2011). Glutamate-vasopressin interactions and the neurobiology of anabolic steroid-induced offensive aggression. *Neuroscience*.

[159] Jenkins B. G. (2012). Pharmacologic magnetic resonance imaging (phMRI): imaging drug action in the brain. *NeuroImage*.

